# Plasma growth differentiation factor‐15 in patients with “lone” atrial fibrillation

**DOI:** 10.1002/jcla.24373

**Published:** 2022-03-25

**Authors:** Na Li, Qian Feng, Fangfang Yu, Jian Zhou, Xueyuan Guo

**Affiliations:** ^1^ Heart Center and Beijing Key Laboratory of Hypertension Beijing Chaoyang Hospital Capital Medical University Beijing China; ^2^ 604064 Department of Third Cardiology Zhangjiakou First Hospital Zhangjiakou China; ^3^ Department of Radiology Beijing Chaoyang Hospital Capital Medical University Beijing China; ^4^ Department of Cardiology Beijing Anzhen Hospital Beijing Institute of Heart Lung and Blood Vessel Diseases Capital Medical University Beijing China

**Keywords:** atrial fibrillation, biomarker, growth differentiation factor‐15

## Abstract

**Background:**

Growth differentiation factor‐15 (GDF‐15) is a member of the transforming growth factor β superfamily, correlated with various stimuli, including cardiovascular disease. The association between plasma GDF‐15 level and “lone” AF, that is, AF of unknown etiology (UeAF), is uncertain.

**Methods:**

All patients aged 60 years or younger. AF patients were hospitalized for primary catheter ablation. Patients with sinus rhythm admitted for other diseases during the same period were included in the control group. ELISA was used to measure plasma GDF‐15 concentrations.

**Results:**

60 UeAF patients, 60 paroxysmal AF (PAF) patients, and 70 control patients were enrolled. The mean age was 44.6 years. In the UeAF group, no patients had traditional clinical risk factors. The plasma GDF‐15 level in the UeAF group was (1028.5 ± 180.5) pg/ml, higher than in the control group, and moderately lower than in the PAF group. In all patients, positive correlations were found between plasma GDF‐15 level and age (*R* = 0.210, *p* < 0.05), and between plasma GDF‐15 level and left atrial diameter (LAD; *R* = 0.338, *p* < 0.05; in the UeAF group: *R* = 0.475, *p* < 0.05; in the PAF group: *R* = 0.504, *p* < 0.05).

**Conclusions:**

Our study first investigated the role of GDF‐15 in UeAF. The plasma GDF‐15 level in UeAF patients was higher than in sinus rhythm patients and lower than in PAF patients. Moreover, GDF‐15 was positively correlated with age and LAD. The role of GDF‐15 in UeAF needs further study.

## Graphical abstract

Like common atrial fibrillation (AF) patients, AF patients without traditional risk factors also had high plasma GDF‐15 level. Moreover, GDF‐15 was positively correlated with left atrial diameter.

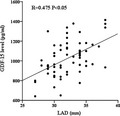

## 1 INTRODUCTION

Growth differentiation factor‐15 (GDF‐15) is a member of the transforming growth factor β superfamily.^1^ Its expression is correlated with diverse stimuli, including oxidation, inflammation, hypoxia, tissue injuries, and so on.^2^, ^3^ Cardiovascular disease is also closely related to GDF‐15 production.^4^ As a popular cardiovascular disease, atrial fibrillation (AF) poses high burden globally. Moreover, about five million new cases are diagnosed annually. Thus, substantial efforts are made toward the mechanism and treatment of AF.^5^ Studies found that plasma GDF‐15 level is high in patients with AF.^6^, ^7^ GDF‐15 involves in left atrial remodeling and acts as a predictor of AF recurrence after ablation.^8^ Moreover, GDF‐15 is related to left atrial/left atrial appendage thrombus^9^ and predicts all‐cause mortality risk in patients with AF.^10^

A subtype of AF that develops in young patients without traditional clinical risk factors, such as hypertension, coronary artery disease (CAD), and cardiomyopathy, has been termed “lone” AF.^11^ Recently, the term has been questioned for the variability of definitions.^12^ Although criticized for its heterogeneous definitions, this type of “untraditional” AF patients is really existed in our real world and the mechanism is not well elucidated.^13^, ^14^ In our study, we have called this cohort “AF of unknown etiology” (UeAF), to distinguish this group from the AF patients with traditional risk factors. Understanding the pathogenesis underlying this disease is of great significance for the prevention.

GDF‐15 plays an important role in the occurrence and development of AF. However, the role of GDF‐15 in UeAF is still unknown. The aim of this study was to evaluate the association of GDF‐15 with UeAF.

## 2 METHODS

### 2.1 Study patients

All patients aged 60 years or younger, and were recruited from our hospitals between January 2020 and June 2021. All AF patients were hospitalized for primary catheter ablation, and echocardiography was performed before catheter ablation. The diagnosis criteria of UeAF were AF without known risk factors, including hypertension, CAD, cardiomyopathy, heart failure, valvular heart disease, uncontrolled thyroid disease, diabetes mellitus, chronic obstructive pulmonary disease (COPD), and obstructive sleep apnea.^13^, ^14^, ^15^ To minimize the influence of the duration of AF, only patients with paroxysmal AF (PAF) were included in our study. For comparison, patients with sinus rhythm who were admitted for other diseases during the same period were included in the control group, and patients with PAF worked as another control group.

Patients were excluded as follows: age >60 years, persistent AF, acute heart failure (New York Heart Association class III or IV), valvular heart disease, congenital heart disease, acute coronary syndrome, prior open heart surgery, severe arrhythmia, malignant tumor, and severe inflammatory disease. The study was approved by the Institutional Research Boards of Beijing Chaoyang Hospital and Zhangjiakou First Hospital and was carried out according to the principles of the Declaration of Helsinki. Written informed consents were obtained from all participants.

### 2.2 Clinical data collection

Demographic and clinical data of all patients were collected by reviewing the medical records, including age, sex, body mass index (BMI), smoking, medical history of hypertension, CAD, heart failure, diabetes mellitus, COPD, and family history of AF. Family history of AF was defined as the occurrence of AF in a first‐degree relative (parent or sibling) at age 65 years or younger, and before the onset of the case in the patient.^16^ In addition, echocardiographic parameters such as left atrial diameter (LAD) and left ventricular ejection fraction (LVEF) were collected.

### 2.3 Collection of blood

Blood was collected from the periphery vein on the second day after admission to the hospital and stored in an anticoagulant tube. Blood samples were centrifuged at 1800 *g* for 15 min, and the supernatant was stored at −80℃ until analysis.

### 2.4 Measurement of GDF‐15 concentrations

Plasma GDF‐15 concentrations were assayed using enzyme‐linked immune sorbent assay kits (Cloud‐Clone Corp, Inc.) according to the manufacturer's instructions. The color intensity was measured with a multi‐well spectrophotometer (BioTek) at 450 nm. Each sample was analyzed in triplicate, and the concentration of each sample was determined as average of the triplicates.

### 2.5 Statistical analysis

Normality was tested using the Kolmogorov–Smirnov test, and a *p* value > 0.05 was defined as normally distributed data. Continuous variables of a normal distribution were expressed as mean ± standard deviation. One‐way ANOVA (multiple groups) and Student's *t* test (group pairs) were carried out for comparisons. Continuous variables of a non‐normal distribution were expressed as median ± quartile ranges. Mann–Whitney *U* test or the Kruskal–Wallis test was used for comparisons. Categorical variables were presented as numbers (percentages) and tested using the Chi‐square test among groups. The Spearman's correlation test was used to calculate the associations between plasma GDF‐15 and other clinical parameters. Two‐sided *p* < 0.05 indicated statistical significance. SPSS 17.0 software (SPSS Inc.) was used for all data analysis.

## 3 RESULTS

### 3.1 Baseline characteristics

Sixty UeAF patients, 60 PAF patients, and 70 control patients with sinus rhythm were enrolled in the study. The baseline characteristics of all patients are listed in Table 1. The patients were relatively young, and the mean age was 44.6 years. No significant differences were found in age, sex, BMI, or smoking among the three groups. In the UeAF group, no patients had hypertension, diabetes mellitus, CAD, heart failure, or COPD. No significant differences were found in comorbidities between the control group and the PAF group, except that more patients with CAD were found in control group (*p* < 0.05). There was no significant difference in the incidence of family history of AF between the two AF groups. Regarding echocardiographic parameters, the mean LAD in the UeAF group was (32.2 ± 2.8) mm, which was the lowest among the three groups.

**TABLE 1:** Baseline characteristics of enrolled patients

Variables	UeAF (*n* = 60)	PAF (*n* = 60)	Control (*n* = 70)
Age (years)	43.2 ± 7.6	44.9 ± 9.3	45.5 ± 6.6
Male, *n* (%)	40 (66.7)	36 (60.0)	42 (60.0)
BMI (kg/m^2^)	25.9 ± 3.1	25.3 ± 3.5	26.3 ± 3.7
Smoking, *n* (%)	22 (36.7)	18 (30.0)	20 (28.6)
Hypertension, *n* (%)	0	35 (58.3)	45 (64.3)
Diabetes mellitus, *n* (%)	0	16 (26.7)	14 (20.0)
CAD, *n* (%)	0	12 (20.0)*	34 (48.6)
Heart failure, *n* (%)	0	8 (13.3)	9 (12.9)
COPD, *n* (%)	0	4 (6.7)	3 (4.3)
Family history of AF, *n* (%)	8 (13.3)	7 (11.7)	–
Echocardiographic parameters			
LAD (mm)	32.2 ± 2.8*^‡^	40.9 ± 7.7*	36.7 ± 6.9
LVEF (%)	65.6 ± 4.7*^‡^	60.7 ± 10.4	62.3 ± 10.2

Note

Data are expressed as *n* (%) or mean ± standard deviation.

Abbreviations: BMI, body mass index; CAD, coronary artery disease; COPD, chronic obstructive pulmonary disease; LAD, left atrial diameter; LVEF, left ventricular ejection fraction; PAF, paroxysmal atrial fibrillation; UeAF, atrial fibrillation of unknown etiology; –, unrecorded.

*p < 0.05 versus Control; ^‡^ *p* < 0.05 versus PAF.

### 3.2 Plasma GDF‐15 level in patients with AF

**FIGURE 1:**
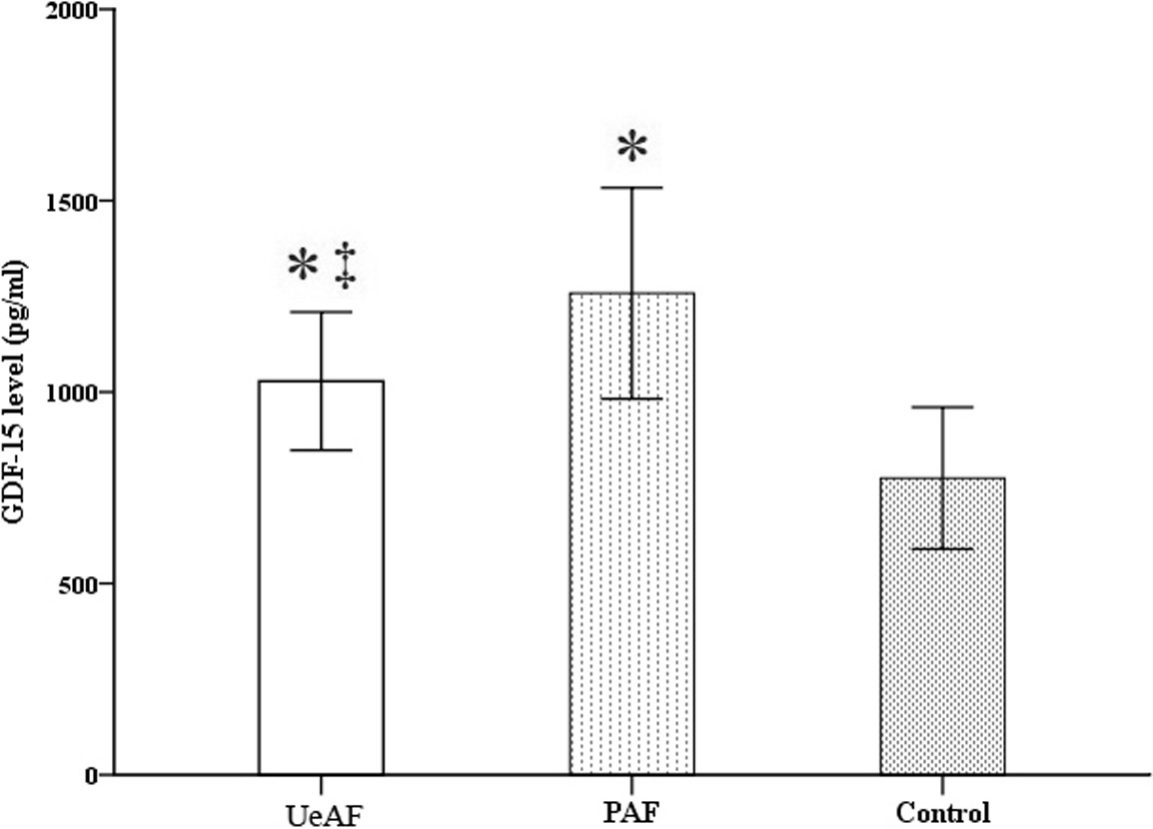
Plasma GDF‐15 level in three groups. *p < 0.05 versus Control; ^‡^ *p* < 0.05 versus PAF

As illustrated in Figure 1, baseline plasma GDF‐15 level in the UeAF group was (1028.5 ± 180.5) pg/ml, which was significantly higher than that in the control group [(775.0 ± 185.2) pg/ml, *p* < 0.05], and was moderately lower than that in the PAF group [(1258.1 ± 275.6) pg/ml, *p* < 0.05]. Among the three groups, the PAF group had the highest level of GDF‐15 (*p* < 0.05).

### 3.3 Correlation of GDF‐15 with clinical parameters

**FIGURE 2:**
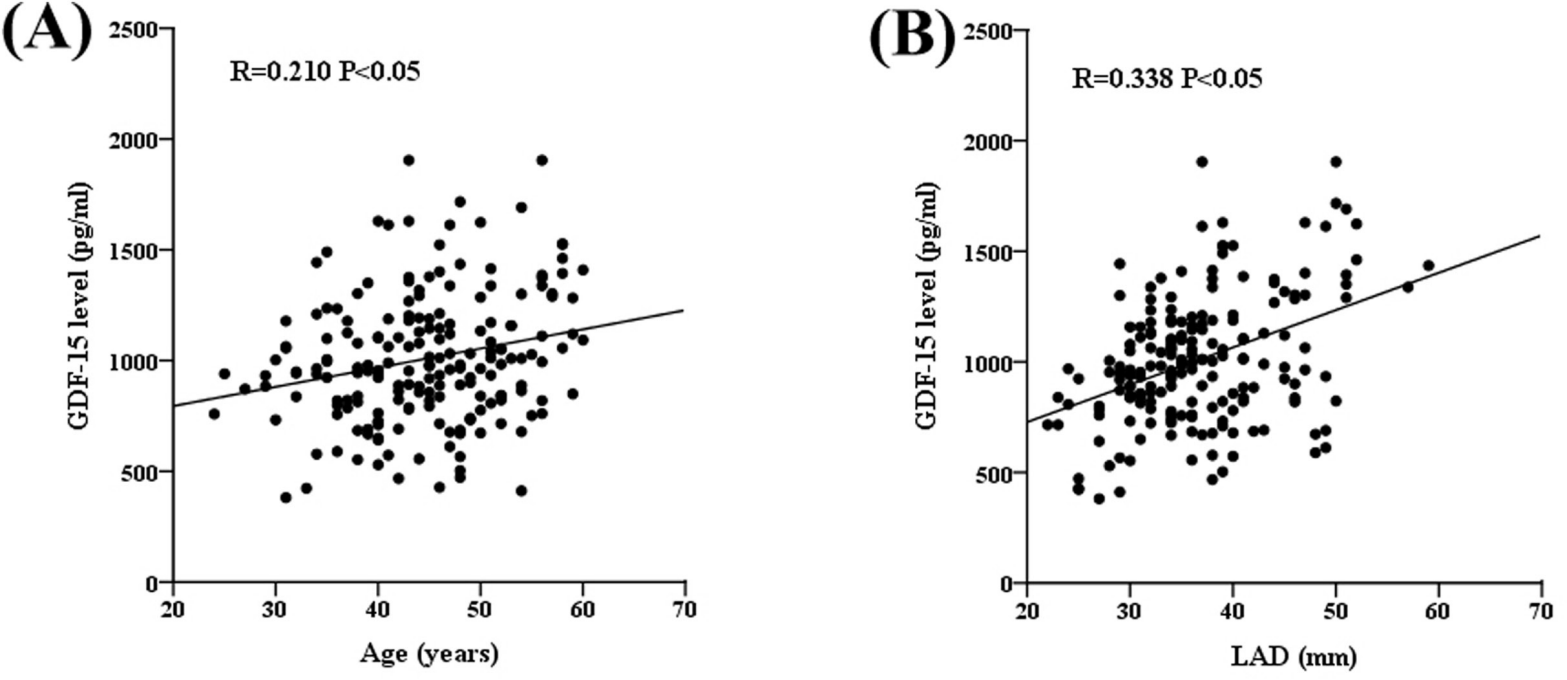
Relationship between plasma GDF‐15 level and baseline characteristics in all patients. (A) A positive correlation between plasma GDF‐15 level and age. (B) A positive correlation between plasma GDF‐15 level and LAD

Spearman's correlation test was used to calculate associations between plasma GDF‐15 and clinical parameters. A positive correlation was found between plasma GDF‐15 level and age in all patients (*R* = 0.210, *p* < 0.05; Figure 2A). Furthermore, an obvious correlation was observed between plasma GDF‐15 level and LAD (*R* = 0.338, *p* < 0.05; Figure 2B). However, no significant correlation was found between plasma GDF‐15 level and BMI or LVEF (*p* > 0.05).

**FIGURE 3:**
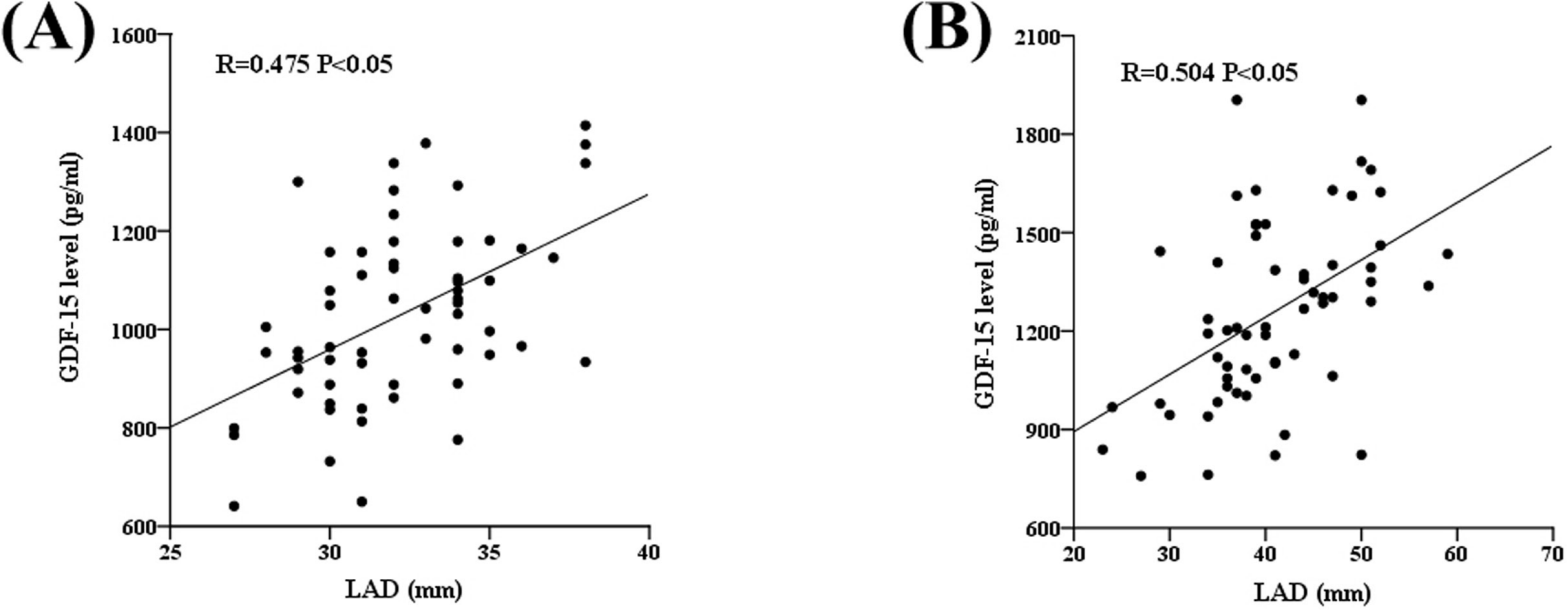
Relationship between plasma GDF‐15 level and LAD in atrial fibrillation patients. (A) The correlation in atrial fibrillation of unknown etiology group. (B) The correlation in paroxysmal atrial fibrillation group

The association between plasma GDF‐15 level and LAD was further analyzed according to the AF type. In the UeAF group, the correlation coefficient between plasma GDF‐15 level and LAD was 0.475 (*p* < 0.05; Figure 3A), and in the PAF group, the correlation coefficient was 0.504 (*p* < 0.05; Figure 3B).

## 4 DISCUSSION

This prospective study investigated the association between baseline plasma GDF‐15 level and AF without traditional risk factors. Our results indicated that baseline GDF‐15 level was higher in UeAF patients than in sinus rhythm patients. Unlike traditional AF patients, UeAF patients did not have traditional risk factors, such as hypertension, CAD, and diabetes mellitus, and the left atrium was not enlarged. We found that the GDF‐15 level was lower in the UeAF group than in the PAF group. Little attention was paid to AF without traditional risk factors, and in our study, it was called UeAF. Therefore, to our knowledge, this is the first study to investigate the role of GDF‐15 in this special type of AF.

AF is a common cardiac arrhythmia, increases with advancing age, and poses high medical burden globally.^17^ The mechanism causing and sustaining AF is complicated. In fact, no unique mechanisms have been identified. Generally, AF is associated with structural heart disease. When structural and/or electrophysiological abnormalities alter atrial tissue, abnormal impulse is formed and/or propagated. In some patients without recognized structural heart disease, inflammatory infiltration and/or fibrosis are detected.^18^, ^19^

“Lone” AF is a special subtype of AF, and it has been applied to young patients without traditional clinical risk factors.^11^ However, definitions are variable between different studies, and with improved identification of predisposing risk factors, the term has fallen out of favor.^20^ Moreover, guidelines suggested that the term should not be used to guide therapy.^21^ In clinical practice, some AF patients are young, without traditional risk factors. Although the term “lone” AF is not popular, this kind of patients also deserves the attention of the researcher. Wijesurendra et al.^13^ enrolled 53 patients with symptomatic paroxysmal or persistent AF and without traditional risk factors for AF, and found that these AF patients have impaired myocardial energetics and subtle left ventricular dysfunction. Though with no obvious structural heart disease, the structure and metabolism of myocardium altered. Canpolat et al.^22^ enrolled 41 symptomatic PAF patients without traditional risk factors and found that a considerable number of the patients (65.9%) exhibit LA fibrosis as determined by cardiac magnetic resonance imaging with delayed enhancement technique (DE‐MRI). Cardiac DE‐MRI is an effective method for noninvasively detecting and quantifying the extent of LA fibrosis.^23^ Recent knowledge on AF has shown that every patient with AF has a cause,^20^ but actually undetected sometimes. A comprehensive cardiovascular assessment including cardiac DE‐MRI in every young patient with AF is recommended.^24^

In the study of Canpolat et al.,^22^ with the help of cardiac DE‐MRI, there was still 34.1% of the AF patients without common risk factors had no LA fibrosis. This indicates the potential role of genetic factors in the development of AF.^25^ Studies found that family history of AF was present in approximately 8%–15% of AF patients.^14^, ^16^, ^26^ In our study, the incidence of family history of AF was 13.3% in the UeAF group and 11.7% in the PAF group. No significant difference was found between the two AF groups. In fact, family history of AF in our study was self‐reported by the patients, and no comprehensive examination was performed in the family members, so the incidence may be underestimated. Generally, relative to patients without, patients with a family history of AF have earlier onset, less comorbidities and more severe symptoms.^14^, ^26^ Genetic testing in selected patients with familial early‐onset AF may be recommended.^27^

GDF‐15 is widely distributed in most organs in low concentrations,^1^ and expression can be highly regulated by inflammation, oxidative stress, and hypoxia.^3^, ^4^, ^28^, ^29^ Furthermore, previous studies have found that GDF‐15 concentrations increase with age.^8^, ^30^ In this study, we also confirmed that in all enrolled patients, GDF‐15 was positively correlated with age. As is well known, the incidence of AF is increased with age.^17^ The exact role of GDF‐15 in AF needs further study.

Left atrial remodeling is the structural basis of AF. Moreover, left atrium enlargement is common in patients with AF and is an indicator of atrial structural remodeling.^31^ Previous studies have shown that patients with a large left atrium are easily prone to developing AF.^32^, ^33^, ^34^ Studies have found that GDF‐15 is a biomarker of cardiac remodeling.^8^, ^29^, ^35^ In this study, we found that the plasma level of GDF‐15 was positively correlated with LAD (*R* = 0.338, *p* < 0.05). In subgroup analysis, the correlation coefficient between plasma GDF‐15 level and LAD was 0.475 (*p* < 0.05) in the UeAF group, and the correlation coefficient was 0.504 (*p* < 0.05) in the PAF group. In fact, in patients in the UeAF group, the LAD was (32.2 ± 2.8) mm, which was in the normal range. We speculate that before left atrium enlargement, inflammation and fibrosis occur in atrial tissue. Myocardial fibrosis is characterized by the accumulation of extracellular matrix. Matrix metalloproteinase and its tissue inhibitor play a vital role in cardiac extracellular matrix remodeling.^36^, ^37^ Moreover, plasma GDF‐15 level was correlated with matrix metalloproteinase and its tissue inhibitor.^38^ It is speculated that GDF‐15 might participate in atrial structural remodeling through collagen synthesis and transformation.

We also explored the association between GDF‐15 and other clinical parameters, such as BMI and LVEF. Studies proved that BMI is a good predictor of cardiovascular disease.^39^ Patients with high BMI are more likely to develop AF.^40^ However, in this study, we did not find a correlation between plasma GDF‐15 level and BMI. LVEF is an indicator of left ventricular function. Yuan et al.^41^ found that in patients without CAD, GDF‐15 levels in correlation between the pericardial fluid GDF‐15 levels and LVEF were significant. Accordingly, we also detect the association between GDF‐15 and LVEF. However, no correlation was found in patients with AF in our study.

There are some limitations of our study. First, the sample size was small, for the diagnosis of UeAF was rigorous, and more patients are needed to confirm our findings. Second, to eliminate the effect of the duration of AF, only PAF was included. To expand our findings, persistent AF patients and long‐standing persistent AF patients are needed in the future study. Third, this study only provides a correlation conclusion, the potential mechanism of GDF‐15 in UeAF needs to be elucidated.

## 5 CONCLUSIONS

We first investigated the role of GDF‐15 in AF without traditional risk factors. The plasma GDF‐15 level in UeAF patients was higher than that in sinus rhythm patients and lower than that in the PAF group. Furthermore, GDF‐15 was positively correlated with age and LAD. The role of GDF‐15 in the development of UeAF needs further study.

## CONFLICT OF INTEREST

The authors have no conflicts of interest.

## Data Availability

The data supporting the findings of this study can be obtained from the corresponding author upon reasonable request.
